# Supplementary material for: Sargasso Sea bacterioplankton community structure and drivers of variance as revealed by DNA metabarcoding analysis
Source: PeerJ. 2022 Feb 28;10:e12835. doi: 10.7717/peerj.12835 (PMC8893026; doi:10.7717/peerj.12835)
Supplement: Supplemental Information 1 — Categorized (A) by categorical sampling depth (surface vs deep chlorophyll maximum (DCM)); and (B) by oceanographic region. Significant values (α < 0.05) are indicated in bold. H′ = Shannon diversity, S = number of observed operational taxonomic units, E = Heip’s evenness, SSS = South Sargasso Sea, NSS = North Sargasso Sea. [file peerj-10-12835-s001.docx]

**TABLE S1.** Kruskal-Wallis analysis of variance tests comparing *α-*diversity statistics from bacterial communities.

Categorized A) by categorical sampling depth (surface vs. deep chlorophyll maximum (DCM)); and B) by oceanographic region. Significant values (α < 0.05) are indicated in bold. H’ = Shannon diversity, S = number of observed operational taxonomic units, E = Heip’s evenness, SSS = South Sargasso Sea, NSS = North Sargasso Sea. Bermuda samples excluded from analyses.

A

|  | Surface vs DCM (All) | | | | Surface vs DCM (SSS) | | | | Surface vs DCM (NSS) | | | |
| --- | --- | --- | --- | --- | --- | --- | --- | --- | --- | --- | --- | --- |
|  | *X^2^* | *DF* | *P* | *X^2^* | | *DF* | *P* | *X^2^* | | *DF* | *P* |  |
| **H’** | 2.17 | 1.00 | 0.14 | **4.85** | | **1.00** | **0.030** | 1.20 | | 1.00 | 0.27 |  |
| **S** | **5.46** | **1.00** | **0.02** | **11.1** | | **1.00** | **0.001** | 1.20 | | 1.00 | 0.27 |  |
| **E** | 0.01 | 1.00 | 0.93 | 0.05 | | 1.00 | 0.830 | 1.20 | | 1.00 | 0.27 |  |
| **Chao 1** | **4.51** | **1.00** | **0.03** | **8.91** | | **1.00** | **0.003** | 0.83 | | 1.00 | 0.36 |  |

B

|  | All Regions | | | | All Regions  (DCM Only) | | | | All Regions  (Surface Only) | | | |
| --- | --- | --- | --- | --- | --- | --- | --- | --- | --- | --- | --- | --- |
|  | *X^2^* | *DF* | *P* | *X^2^* | | *DF* | *P* | *X^2^* | | *DF* | *P* |  |
| **H’** | 2.88 | 3.00 | 0.41 | 3.97 | | 3.00 | 0.26 | 4.01 | | 3.00 | 0.26 |  |
| **S** | 1.61 | 3.00 | 0.66 | 3.82 | | 3.00 | 0.28 | 6.04 | | 3.00 | 0.11 |  |
| **E** | 3.84 | 3.00 | 0.28 | 3.25 | | 3.00 | 0.36 | 1.72 | | 3.00 | 0.63 |  |
| **Chao 1** | 1.01 | 3.00 | 0.80 | 3.54 | | 3.00 | 0.32 | 4.88 | | 3.00 | 0.18 |  |
