# Supplementary material for: Sargasso Sea bacterioplankton community structure and drivers of variance as revealed by DNA metabarcoding analysis
Source: PeerJ. 2022 Feb 28;10:e12835. doi: 10.7717/peerj.12835 (PMC8893026; doi:10.7717/peerj.12835)
Supplement: Supplemental Information 2 — P-values are indicated in parentheses. S = observed operational taxonomic units, H′ = Shannon diversity, E = evenness. [file peerj-10-12835-s002.docx]

**TABLE S2.** Community dissimilarities between geographic regions based on a two-way SIMPER (similarity percentage) analysis.

All Antilles Current samples (*N* = 3) were taken at the DCM (deep chlorophyll maximum) only. NA = North Atlantic, AC = Antilles Current, NSS = North Sargasso Sea, SSS = South Sargasso Sea.

|  | | | |
| --- | --- | --- | --- |
| **Region 1** | **Region 2** | **Dissimilarity (%)** | **No. OTUs contributing to 10% of dissimilarity** |
| NA | AC | 74.06 | 54 |
| NSS | NA | 65.73 | 64 |
| NSS | AC | 62.40 | 61 |
| SSS | NSS | 70.46 | 56 |
| SSS | NA | 73.39 | 49 |
| SSS | AC | 70.05 | 50 |
