# Supplementary material for: Sargasso Sea bacterioplankton community structure and drivers of variance as revealed by DNA metabarcoding analysis
Source: PeerJ. 2022 Feb 28;10:e12835. doi: 10.7717/peerj.12835 (PMC8893026; doi:10.7717/peerj.12835)
Supplement: Supplemental Information 3 — (A) communities throughout water column compared based on depth and based on region, (B) communities from one portion of the water column compared by region, (C) communities from within the Sargasso Sea compared by region and by depth. SSS = South Sargasso Sea, NSS = North Sargasso Sea. [file peerj-10-12835-s003.docx]

**TABLE S3.** PERMANOVA results comparing bacterial communities by depth (surface and deep chlorophyll maximum (DCM) and oceanographic region (NA = North Atlantic, AC = Antilles Current, NSS = North Sargasso Sea, SSS = South Sargasso Sea).

A) All communities throughout water column compared based on depth and based on region, B) communities from isolated oceanographic regions compared by depth, C) communities from one portion of the water column compared by region, D) pairwise tests of surface communities compared by region, E) pairwise tests of DCM communities compared by region. Significant values (α < 0.05) are in bold.

A

| **All Communities** |  |  | **All Communities** |  |
| --- | --- | --- | --- | --- |
| **By Depth** |  |  | **By Region** |  |
| sample size | 39 |  | sample size | 39 |
| number of groups | 2 |  | number of groups | 4 |
| pseudo-*F* | 0.492 |  | pseudo-*F* | **1.892** |
| *P*-value | 0.832 |  | *P*-value | **0.028** |
| number of permutations | 999 |  | number of permutations | 999 |

B

| **SSS Communities** |  |  | **NSS Communities** |  |  | **NA Communities** |  |
| --- | --- | --- | --- | --- | --- | --- | --- |
| **By Depth** |  |  | **By Depth** |  |  | **By Depth** |  |
| sample size | 21 |  | sample size | 11 |  | sample size | 4 |
| number of groups | 2 |  | number of groups | 2 |  | number of groups | 2 |
| pseudo-*F* | 4.322 |  | pseudo-*F* | **14.73** |  | pseudo-*F* | 0.585 |
| *P*-value | 0.099 |  | *P*-value | **0.004** |  | *P*-value | 1.0 |
| number of permutations | 999 |  | number of permutations | 999 |  | number of permutations | 999 |

C

| **Deep Communities Only** | |  | **Surface Communities Only** | |
| --- | --- | --- | --- | --- |
| **By Region** | |  | **By Region** | |
| sample size | 22 |  | sample size | 17 |
| number of groups | 4 |  | number of groups | 3 |
| pseudo-*F* | **2.765** |  | test statistic | **4.640** |
| *P*-value | **0.003** |  | *P*-value | **0.001** |
| number of permutations | 999 |  | number of permutations | 999 |

D

| **SSS and NSS Surface Communities** |  |  | **SSS and NA Surface Communities** |  |  | **NSS and NA Surface Communities** |  |
| --- | --- | --- | --- | --- | --- | --- | --- |
| **By Region** |  |  | **By Region** |  |  | **By Region** |  |
| sample size | 15 |  | sample size | 11 |  | sample size | 8 |
| number of groups | 2 |  | number of groups | 2 |  | number of groups | 2 |
| pseudo-*F* | **8.252** |  | pseudo-*F* | 1.978 |  | pseudo-*F* | 2.892 |
| *P*-value | **0.002** |  | *P*-value | 0.112 |  | *P*-value | 0.152 |
| number of permutations | 999 |  | number of permutations | 999 |  | number of permutations | 999 |

E

| **SSS and NSS DCM Communities** |  |  | **SSS and AC DCM Communities** |  |  | **SSS and NA DCM Communities** |  |
| --- | --- | --- | --- | --- | --- | --- | --- |
| **By Region** |  |  | **By Region** |  |  | **By Region** |  |
| sample size | 17 |  | sample size | 15 |  | sample size | 14 |
| number of groups | 2 |  | number of groups | 2 |  | number of groups | 2 |
| pseudo-*F* | **4.300** |  | pseudo-*F* | **3.213** |  | pseudo-*F* | 1.360 |
| *P*-value | **0.004** |  | *P*-value | **0.021** |  | *P*-value | 0.253 |
| number of permutations | 999 |  | number of permutations | 999 |  | number of permutations | 999 |

| **NSS and AC DCM Communities** |  |  | **NSS and NA DCM Communities** |  |  | **AC and NA DCM Communities** |  |
| --- | --- | --- | --- | --- | --- | --- | --- |
| **By Region** |  |  | **By Region** |  |  | **By Region** |  |
| sample size | 8 |  | sample size | 7 |  | sample size | 5 |
| number of groups | 2 |  | number of groups | 2 |  | number of groups | 2 |
| pseudo-*F* | **2.741** |  | pseudo-*F* | **3.035** |  | pseudo-*F* | 1.315 |
| *P*-value | **0.039** |  | *P*-value | **0.046** |  | *P*-value | 0.298 |
| number of permutations | 999 |  | number of permutations | 999 |  | number of permutations | 999 |
